# Dependence of phase transition uniformity on crystal sizes characterized using birefringence

**DOI:** 10.1063/4.0000098

**Published:** 2021-06-30

**Authors:** Saminathan Ramakrishnan, Jason R. Stagno, Valentin Magidson, William F. Heinz, Yun-Xing Wang

**Affiliations:** 1 Structural Biophysics Laboratory, Centre for Cancer Research, National Cancer Institute, Frederick, Maryland 21702, USA; 2 Optical Microscopy and Analysis Laboratory, Cancer Research Technology Program, Frederick National Laboratory for Cancer Research, Frederick, Maryland 21702, USA

## Abstract

Solid–solid phase transitions (SSPTs) have been widely observed in crystals of organic or inorganic small-molecules. Although SSPTs in macromolecular crystals have been reported, the majority involve local atomic changes, such as those induced by changes in hydration. SSPTs driven by large conformational changes, however, can be more difficult to characterize since they often significantly disrupt lattice packing interactions. Such drastic changes make the cooperativity of molecular motion at the atomic level less easily achieved and more dependent on intrinsic properties of the crystal that define lattice order. Here, we investigate the effect of crystal size on the uniformity of SSPT in thin plate-like crystals of the adenine riboswitch aptamer RNA (riboA) by monitoring changes in crystal birefringence upon the diffusion of adenine ligand. The birefringence intensity is directly related to molecular order and the concurrent changes to polarizability of molecules that results from structural changes throughout the phase transition. The riboA crystals were loosely grouped into three categories (small, medium, and large) based on the surface area of the crystal plates. The time width of transition increased as a function of crystal size, ranging from ∼13 s for small crystals to ∼40 s for the largest crystal. Whereas the transitions in small crystals (<10 *μ*m^2^) were mostly uniform throughout, the medium and large crystals exhibited large variations in the time and width of the transition peak depending on the region of the crystal being analyzed. Our study provides insight into the spatiotemporal behavior of phase transitions in crystals of biological molecules and is of general interest to time-resolved crystallographic studies, where the kinetics of conformational changes may be governed by the kinetics of an associated SSPT.

## INTRODUCTION

Virtually all intracellular and extracellular events occur through discrete biomolecular interactions. The dynamics and inducible structural changes of these molecules often underpin their functional roles. Over the last several decades, structural biologists have elucidated atomic structures of many of these molecules and their complexes, which have proven invaluable to advancing important areas of biomedical research. In most cases, however, such structural information has been limited primarily to static and binary (on/off, bound/unbound) pictures that attempt to explain a much more dynamic and complex phenomenon. The search for knowledge embedded in conformational changes has sparked a new era of time-resolved X-ray crystallography (TRX), but the burgeoning method has presented its own technical challenges. Some of those have been circumvented by unprecedented technological advances at both synchrotron and X-ray free electron laser (XFEL) sources, which have paved the way for TRX with quick-readout detectors and ultra-fast X-ray pulses at higher intensities.

As with any crystallography experiment, however, the most fundamental criterion for TRX is crystal diffraction quality, and this challenge is compounded by crystals whose contents are changing in real time. The core of the crystallographic method relies on the ordered array of “static” molecules to produce Bragg diffraction. Inducing large conformational changes, therefore, which are likely to disrupt the lattice, seems counterintuitive to making the crystals in the first place. For this reason, the majority of TRX studies have been limited to molecules exhibiting small (sub-angstrom) structural changes that can be accommodated by the lattice environment and pose little detriment to diffraction quality. Large conformational changes in crystals, however, require a certain level of “synchrony” at both the molecular and lattice levels to minimize lattice disorder and to prevent entry into the liquid phase, and the crystal may end up in a different lattice altogether. Such solid–solid phase transitions (SSPTs) may also involve one or more intermediate lattices, as the molecules reorient to achieve more energetically favorable packing. All of these scenarios have not been investigated until recently (Ramakrishnan *et al.*, 2021).

Whether the conformational changes are large or small, the activation of those changes must occur at a rate comparable to or faster than the changes themselves. For photoactivated systems, the time for molecular stimulation is negligible (Kupitz *et al.*, 2014; Pande *et al.*, 2016; Suga *et al.*, 2020; Tenboer *et al.*, 2014). For other biomolecular crystals, however, where the conformational changes are triggered by a ligand, one must first consider the rate of diffusion. Previous experimental and simulated data for crystals of various sizes indicated that very small (0.1–1 *μ*m) nano/microcrystals may have sub-millisecond diffusion times, whereas the diffusion in larger (tens to hundreds of micrometers) crystals are in the time-regime of seconds (Schmidt, 2013). For TRX of small conformational changes, therefore, nano/microcrystals are necessary such that the diffusion time is roughly on a similar timescale as the structural perturbations. In contrast, large conformational changes, particularly those that are restricted by lattice confinement, may take as long as milliseconds to seconds to accumulate—much slower than diffusion times—thus making diffusion negligible.

There is another critical reason, however, which has been largely unexplored, for why nano/microcrystals are essential for TRX experiments involving changes to the crystal lattice—transition uniformity. By virtue of having a larger surface area to volume ratio, smaller crystals are expected to have higher plasticity and to be more amenable to holistic changes to the crystal structure without compromising lattice integrity. Larger crystals, on the other hand, may be less forgiving of such changes. The higher occurrence of defects and nonuniformities within large crystals can result in mosaic subdomains of varying lattice character, which create weak points in the crystal that are more susceptible to mechanical strain. Quake-like events, such as those triggered by conformational changes, typically manifest as cracks, fissures, or crystal fragmentation. Consequently, larger crystals are far less likely to exhibit uniform changes, thus making nano/microcrystals all the more essential and an XFEL beam more suitable for TRX studies involving lattice changes. How small is a lingering topic of discussion that requires further investigation into the uniformity of macromolecular crystal transitions in crystals of various sizes.

SSPTs in both man-made and natural materials have been studied extensively at the macroscopic level for applications in physical sciences and engineering (Commins *et al.*, 2016; Porter *et al.*, 2008). The transformation between crystalline forms of the same material, in both forward and reverse directions, is typically dictated by external physical factors such as temperature, humidity, and pressure (Anwar and Zahn, 2017; Dunitz, 1992). Changes in protein crystal hydration can result in local conformational changes that influence crystal packing interactions, which in turn may result in changes to unit cell constants and, in some cases, crystal symmetry (Berthou and Jolles, 1974; Dobrianov *et al.*, 2001; Huxley and Kendrew, 1953; Klingl *et al.*, 2015; Kodandapani *et al.*, 1990; Nagendra *et al.*, 1998; Perutz, 1946; Pinard *et al.*, 2016; Salunke *et al.*, 1985). Though SSPTs in organic and inorganic materials have been reported previously, elucidating the mechanisms of SSPT at the atomic and molecular levels still requires reliable methodology and high-throughput screening (Khaliullin *et al.*, 2011; Peng *et al.*, 2015; Pogatscher *et al.*, 2016; Smets *et al.*, 2020; Taniguchi *et al.*, 2019). Optical microscopy studies of SSPT in small organic crystals or colloids lack spatiotemporal information and do not necessarily support the mechanisms proposed by Ehrenfest (Mnyukh, 2009; Mnyukh and Panfilova, 1973). The concerted angstrom-scale motion of molecules in a crystal—called a diffusionless martensitic transition—occurs at the speed of sound, whereas a displacive transition involves long-range molecular movements in the crystal during SSPT. One approach to characterize SSPT is the use of polarized video microscopy (PVM) to monitor changes in crystal birefringence (Horie *et al.*, 2007, 2012). Birefringence is observed in ordered organic and inorganic materials due to differences (anisotropy) in the refractive index as a function of molecular orientation. Since many biological molecules are optically anisotropic, polarized optical microscopy has been used to study protein assembly (Vrabioiu and Mitchison, 2006), cell division (LaFountain and Oldenbourg, 2004), cellular contractility (Wang *et al.*, 2018), high-throughput imaging of biological specimens (Shribak, 2015), and molecular orientation in biological filaments (Cruz *et al.*, 2016). In optically anisotropic crystalline materials, birefringence is observed due to an unequal behavior of refracted light from different cell axes, which changes with crystal symmetry (Mehta *et al.*, 2013). The unique double-refraction of light—or crystal birefringence—is used for direct spatiotemporal observation of changes. In materials science, polarized optical microscopy has also been used to characterize polymers and liquid crystals, particularly their morphological features in the normal state vs during or after a phase transition (Choi *et al.*, 2004; Gim *et al.*, 2017; Yoon *et al.*, 2007).

For the proper characterization of SSPT in biomolecular crystals, we used crystals of the adenine riboswitch aptamer domain (riboA). Riboswitches are highly structured RNA molecules that function as genetic control elements through conformational switching in response to specific cellular metabolites (Nahvi *et al.*, 2002). The riboA molecules undergo large conformational changes upon the binding of adenine, resulting in distinct and measurable lattice changes from monoclinic (apo unit cell, AUC) to triclinic (trans unit cell 1, TUC1) to orthorhombic (bound unit cell, BUC) (Stagno *et al.*, 2017). Previous studies using XFEL and atomic force microscopy (AFM) demonstrated that the ligand-induced SSPT in riboA crystals is continuous and reversible and involves concerted large conformational changes that alter crystal packing interactions (Ramakrishnan *et al.*, 2021). However, the propagation speed of SSPT in riboA crystals is ligand-concentration- and temperature-dependent. Here, we show that the SSPT is also crystal-size dependent and that larger crystals exhibit a significantly larger degree of nonuniformity of transition than small crystals in terms of transition time width. These results are highly informative for future TRX studies in that very small crystals may be essential for determining time-resolved conformations.

## RESULTS AND DISCUSSION

**FIG. 1.**
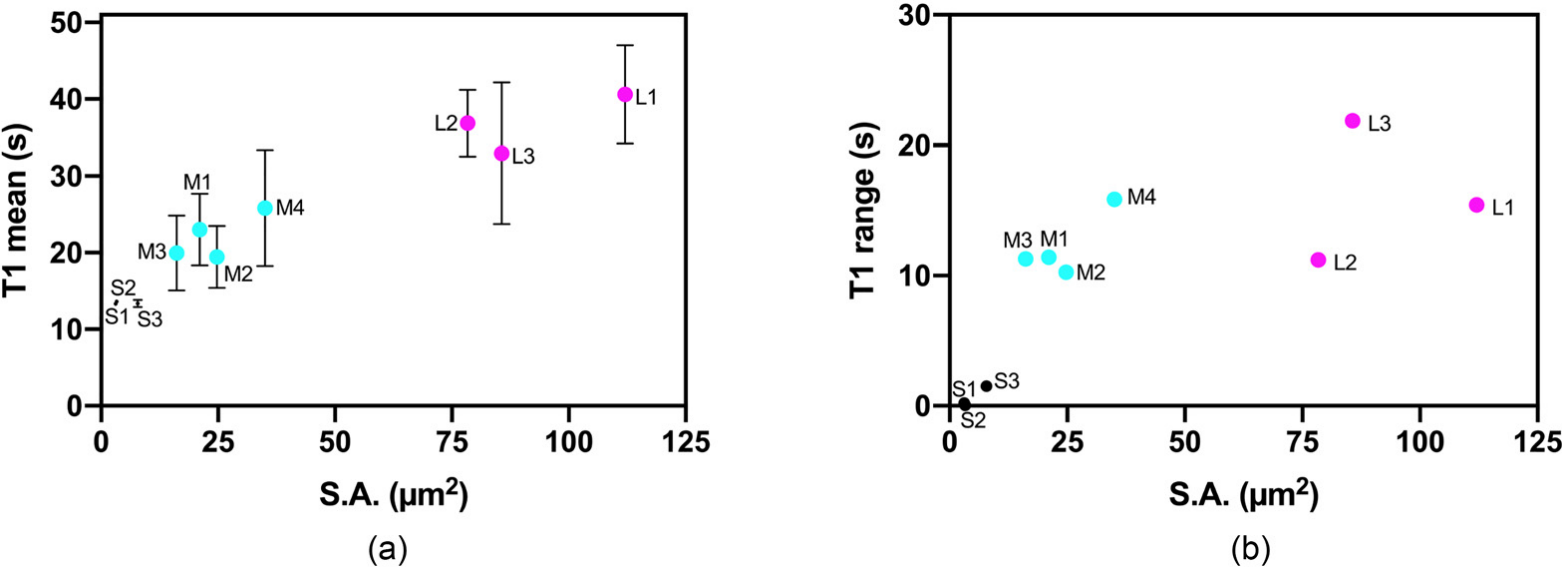
(a) Mean transition time (T1) and (b) range (difference between maximum and minimum values observed) of T1 for the 7 ROIs measured for the 10 different riboA crystals of various sizes (small: S1, S2, S3, black; medium: M1, M2, M3, M4, cyan; large: L1, L2, L3, magenta). The size of each crystal is represented by its observable surface area (SA).

To investigate the effect of crystal size on the spatiotemporal behavior of the phase transition, we analyzed changes in crystal birefringence intensity for 10 crystals with plate dimensions ranging from 1.2 to 11.9 *μ*m at a ligand concentration of 10 mM (Fig. 1, supplementary material). Throughout the phase transition, the birefringence of the crystal decreases as a result of the ligand-induced conformational changes that reorient the molecules and their respective crystallographic axes (Movies 1–3, supplementary material). This characteristic behavior of the crystals under polarized light was then used to identify the onset and speed of phase transition upon ligand addition. Our custom-built MATLAB code quantifies the range of intensity for a selected region of interest (ROI), for a given spatial binning size, and derives the first-order expression or rate of intensity change as a function of time. The crystals were categorized according to size: small (S1, S2, S3), medium (M1, M2, M3, M4), and large (L1, L2, L3) (Table I). As the crystals are thin plates that lay flat on the glass surface, the crystal thickness could not be measured for each, but is estimated to be < 1 *μ*m, and could vary slightly from crystal to crystal. For simplicity, therefore, the sizes of the crystals are described by their surface areas (SAs) (Table I).

**TABLE I.** Crystal dimensions and mean transition time (T1) with standard deviation for the 10 riboA crystals: S1–S3 (small), M1–M4 (medium), and L1–L3 (large).

Crystal	Dimensions (*μ*m)	Surface Area (*μ*m^2^)	Mean T1 (s)
S1	2.6 × 1.2	3.1	13.34 +/– 0.07
S2	2.2 × 1.5	3.3	13.60 +/– 0.01
S3	3.5 × 2.2	7.7	13.38 +/– 0.48
M1	5.2 × 4.1	21.3	23.03 +/– 4.68
M2	6.7 × 3.7	24.8	19.44 +/– 4.03
M3	5.3 × 3.0	15.9	19.96 +/– 4.89
M4	7.6 × 5.3	40.3	25.81 +/– 7.59
L1	11.1 × 10.1	112.1	40.59 +/– 6.45
L2	9.8 × 7.8	76.4	36.85 +/– 4.34
L3	11.9 × 7.2	85.7	32.96 +/– 9.23

**FIG. 2.**
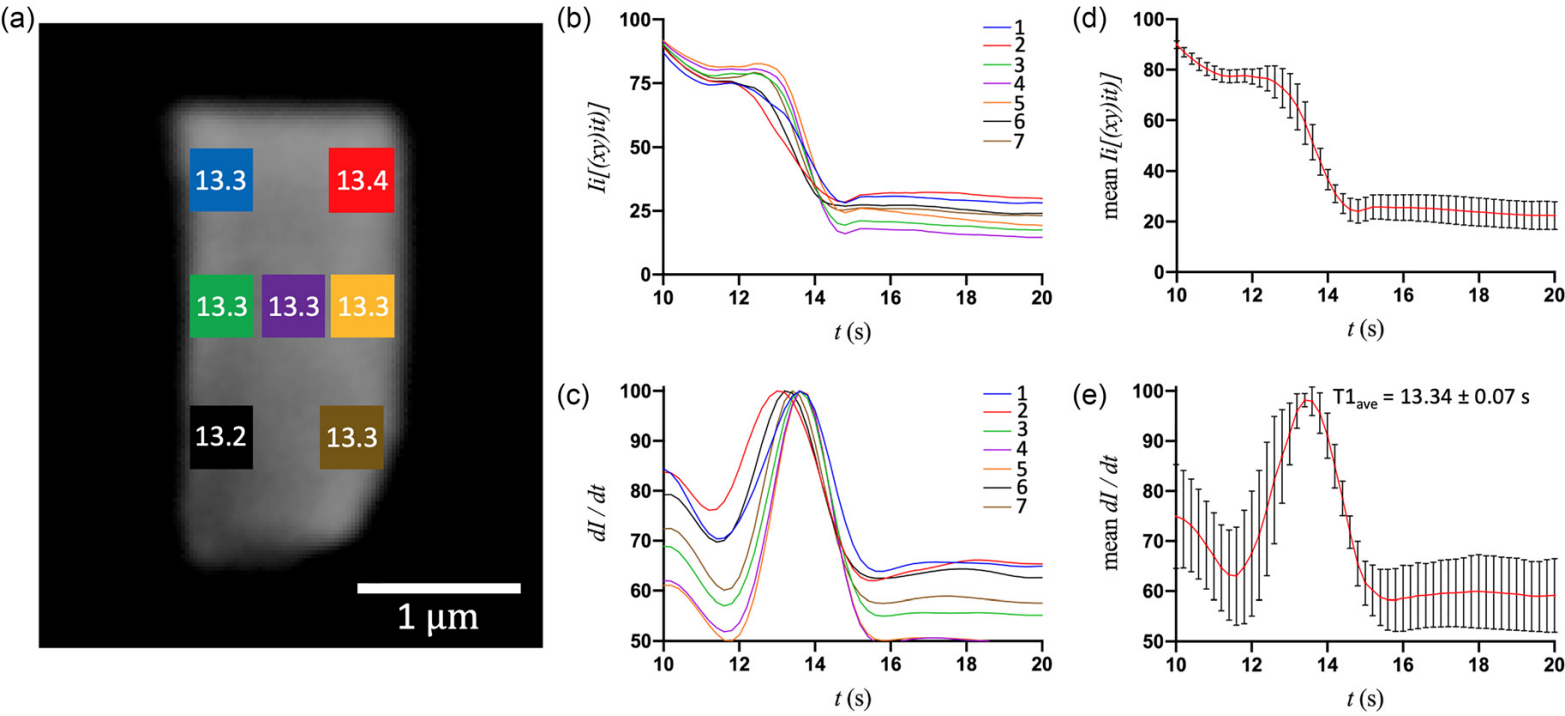
(a) The optical microscope image of a small (2.6 × 1.2 *μ*m^2^) crystal (S1) with the seven different ROIs (20 x 20 pixels^2^ each) and their respective T1 values. (b) and (c) Corresponding (by color) intensity and first-derivative curves of the middle pixel of each ROI after the addition of 10 mM adenine ligand. (d) and (e) Mean values (with error bars) of intensity and first-derivative curves for all seven ROIs, plotted as a function of time.

The effect of crystal size on the phase transition was first examined by comparing the time of transition (T1) for seven ROIs (20 × 20 pixels^2^) representing different regions of the crystal. Based on our previous studies, the conformational switching of the riboswitch occurs T1 seconds after ligand addition. Thus, the spatiotemporal characteristics of the crystals during the transition are essential to the RNA's structural dynamics. The mean values of T1 clearly indicate that the onset of transition increases as a function of crystal size [Fig. 1(a), Table I]. More importantly, the range variation in the T1 values observed at different regions of the crystal is quite large for medium and large crystals, but almost negligible for small crystals [Fig. 1(b) and Table I; Fig. 2, supplementary material].

**FIG. 3.**
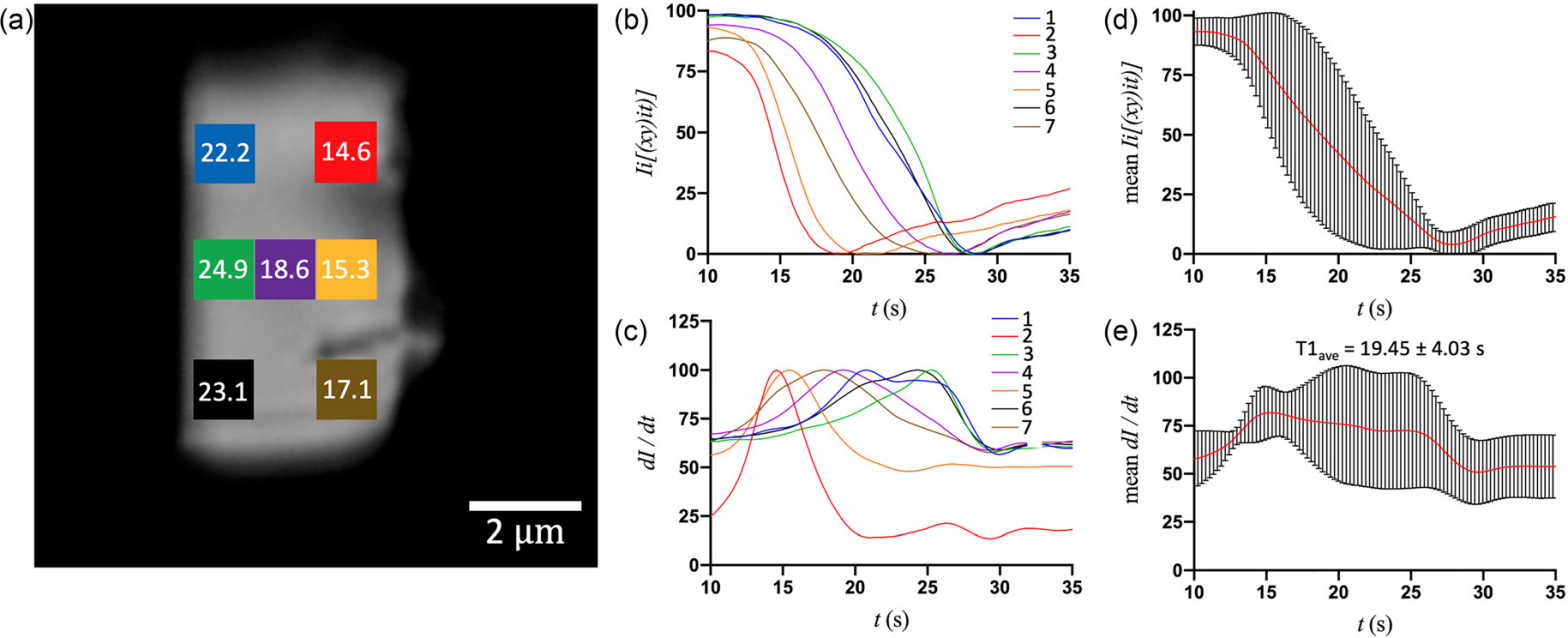
(a) The optical microscope image of a medium (6.7 × 3.7 *μ*m^2^) crystal (M2) with the seven different ROIs (20 × 20 pixels^2^ each) and their respective T1 values. (b) and (c) Corresponding (by color) intensity and first-derivative curves of the middle pixel of each ROI after the addition of 10 mM adenine ligand. (d) and (e) Mean values (with error bars) of intensity and first-derivative curves for all 7 ROIs, plotted as a function of time.

Figure 2(a) shows the optical microscopy image of a representative small crystal (S1) and derived T1 values for each of the seven ROIs. The birefringence intensity [Fig. 2(b)] and first-derivative curves [Fig. 2(c)] of the middle pixel of each ROI show that there is uniformity in the transition kinetics at all places in the crystal. Moreover, plotting the average intensity and first-derivative curves [Figs. 2(d) and 2(e)] reveals that the standard deviation is comparatively lowest at the transition peak (T1), which occurs at 13.2–13.4 s throughout the crystal. Though the top-right and bottom-left regions of the crystal show a minor deviation in T1 (0.1–0.2 s) relative to the middle of the crystal, the phase transition in the small crystals is evidently uniform, indicating a level of synchrony in the molecular conformational changes and lattice rearrangement (Figs. 1 and 2). The medium crystals, on the other hand, show much greater variation in T1 at different regions (Fig. 3; supplementary material). For crystal M2, the transition originates from the top-right at ∼14.6 s and proceeds gradually to the left with the latest T1 value occurring at ∼24.9 s. Therefore, an increase in crystal size by only a few micrometers on each edge introduces a heterogeneity of ∼10 s in the time of transition. The single-pixel intensity and first-derivative curves of M2 exhibit an incremental delay from the right side of the crystal to the left [Figs. 3(a)–3(c)], and the averages of these curves have large standard deviations, particularly at the transition peaks [Figs. 3(d) and 3(e)], in stark contrast to crystal S1 [compare with Figs. 2(d) and 2(e)].

**FIG. 4.**
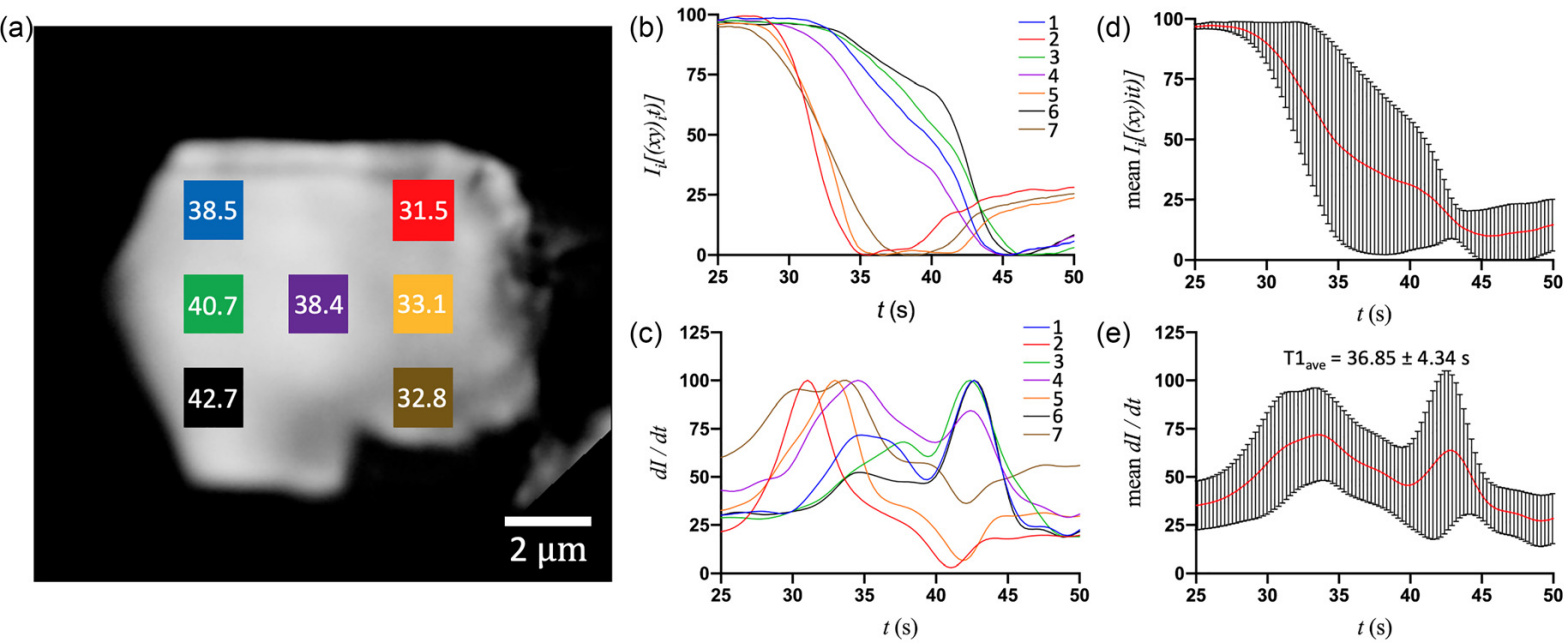
(a) The optical microscope image of a large (9.8 × 7.8 *μ*m^2^) crystal (L2) with the seven different ROIs (20 × 20 pixels^2^ each) and their respective T1 values. (b) and (c) Corresponding (by color) intensity and first-derivative curves of the middle pixel of each ROI after the addition of 10 mM adenine ligand. (d) and (e) Mean values (with error bars) of intensity and first-derivative curves for all seven ROIs, plotted as a function of time.

The large crystals also exhibited a significant location-dependent variation in transition time between the left, middle, and right regions of the crystal (Fig. 4; Fig. 4, supplementary material). Similar to M2, the transition in crystal L2 originates from the top-right (∼31.5 s) and progresses toward the bottom-left (∼42.7 s) [Fig. 4(a), red and black squares], with a variation in T1 of ∼11 s. Since L2 is larger than M2 in both dimensions (8 × 9.8 *μ*m^2^ vs 6.7 × 3.7 *μ*m^2^), a similar variation in T1 indicates that the propagation of transition throughout the crystal is slightly faster in L2. Notably, although the time of transition increases with crystal size [Fig. 1(a)], the range in T1 values is comparable for the medium and large crystals and shows no discernible correlation, only that they are non-uniform [Fig. 1(b)].

**FIG. 5.**
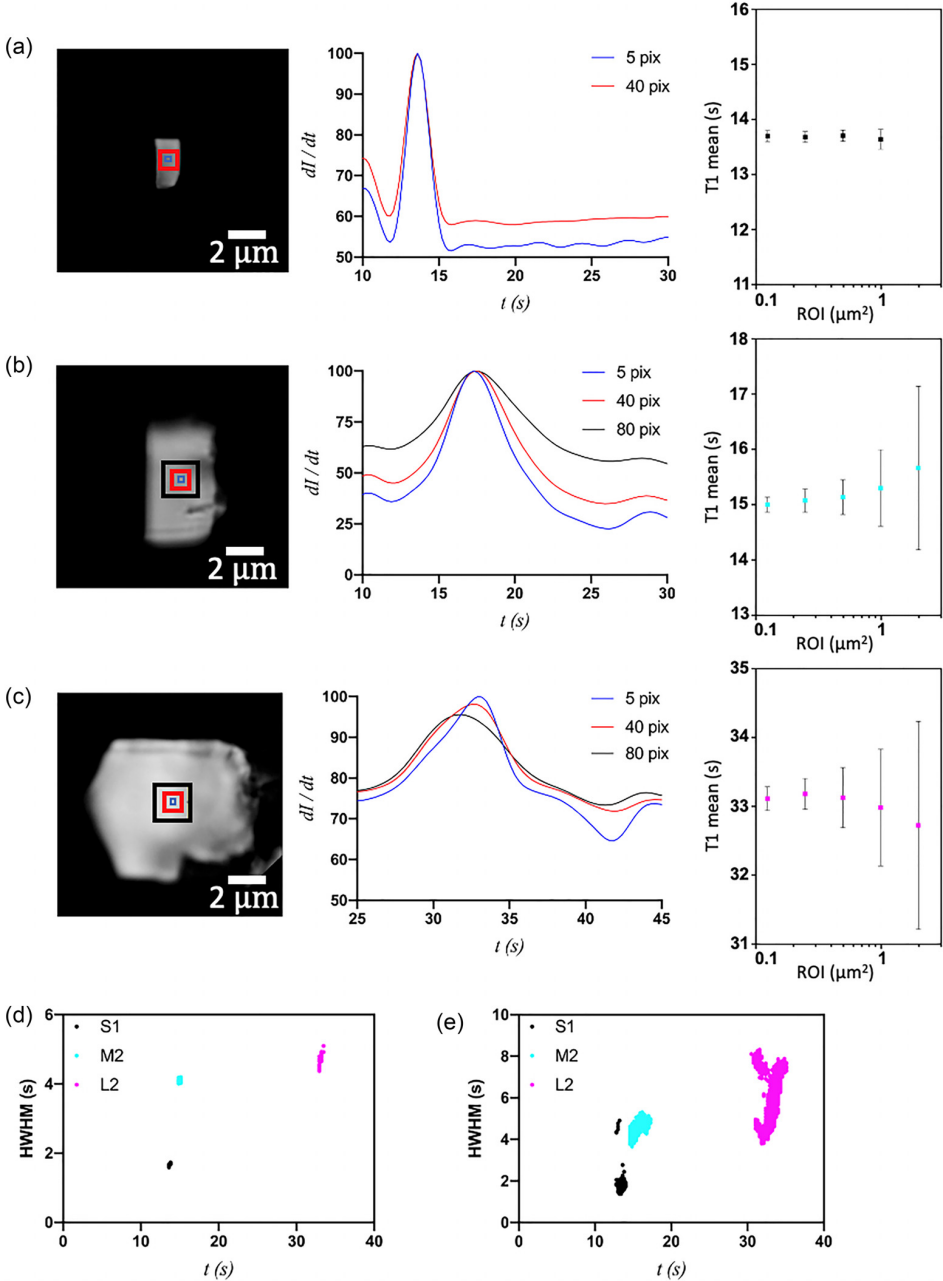
Optical microscopic images of S1 (a), M2 (b), and L2 (c) crystals, with concentric ROIs (red boxes) with sizes of 5, 40, or 80 pixels (number of pixels of the ROI edge); first-derivative of birefringence intensity as a function of time (*dI*/*dt*) for S1 (5, 40 pixels), M2 (5, 40, 80 pixels), and L2 (5, 40, 80 pixels); and T1 mean values (with error bars for the standard deviation) plotted as a function of ROI size. (d) and (e) Plot of transition width (peak width at half-max, PWHM) vs time for S1 (black), M2 (cyan), and L2 (magenta) for ROI sizes of 5 pixels (d) and 40 pixels (e).

As an alternate method for demonstrating the differences in the phase transition kinetics between crystals S1, M2, and L2, the birefringence intensity profiles were measured for ROIs of increasing size, centered on the same pixel (concentric) (Fig. 5). Of note, these three crystals were chosen for analysis because they exhibited the least amount of motion on the glass surface during the phase transition, thus minimizing the influence of positional variation in the selected region and making these crystals most amenable for detailed characterization and analysis. The widths of the ROIs were 5, 10, 20, and 40 pixels for all three crystals, as well as 80 pixels for M2 and L2. As expected for the small crystal, whose transition is virtually uniform throughout, changing the size of the ROI being analyzed showed very little difference in T1 [Fig. 5(a); supplementary material]. In the medium and large crystals, however, the derived value for T1 changes with respect to ROI size. Moreover, the standard deviation of T1 increases almost linearly with ROI size for M2 and L2 [Figs. 5(b) and 5(c); supplementary material]. In contrast, the standard deviation of T1 is constant for all ROIs except 40 pixels. However, even within a crystal area of 1 *μ*m^2^ (40 × 40 pixels^2^), the standard deviation of T1 at the pixel level in the small crystal is only 0.18 s, whereas that same square area in a medium or large crystal exhibits T1 standard deviations of 0.69 and 0.85 s, respectively. In addition, the transition peak half-width at half-max (HWHM) for a given ROI, which measures the spread of non-uniformity of the transition within the selected region, increases with crystal size (supplementary material). Such differences may result, in part, from crystal motion or differences in crystal thickness, but the influence of such effects does not alter the qualitative interpretations that the transitions in larger crystals exhibit greater heterogeneity overall.

## CONCLUSION

Our study provides spatiotemporal characterization of molecular phase transitions in crystals of various sizes in real-time. PVM birefringence intensity data were recorded for 10 crystals of riboA with dimensions ranging from 1.2 to 11.9 *μ*m. Two sets of analyses were performed that investigated the effect of crystal size on the time and uniformity of the ligand-induced phase transition. The first analysis compared the intensity data for a single bin size (20 × 20 pixels^2^) at different locations in each crystal, whereas the second analysis compared data of varying bin sizes centered around a single location. Both analyses consistently demonstrated that the phase transition in riboA crystals driven by adenine ligand at 10 mM is not only size dependent but also non-uniform for crystals larger than 10 *μ*m^2^. It is clear that T1 increases as a function of crystal size. Given that the time for ligand diffusion is expected to be on the scale of milliseconds for even the largest crystals examined (Schmidt, 2013), the effect of diffusion on the spatiotemporal behavior of the phase transition is likely negligible. Therefore, the relationship between T1 and crystal size indicates a variation in the time and ability for enough molecules to reach an energy state capable of overcoming the energy imposed by lattice restraints. Reaching such an energy state would be, of course, more easily achieved in crystals that exhibit greater lattice order and molecular uniformity before and during the phase transition. The small crystals (<10 *μ*m^2^) analyzed in this study seem to possess these properties, exhibiting the earliest and most uniform transition throughout the crystal. In contrast, crystals >10 *μ*m^2^ showed spatial dependencies on both the time and width of the transition.

Although the effects of crystal thickness could not be accounted for, it is reasonable to expect that an increase in the crystal dimension of *z* would exhibit the same effects as those observed for *x* and *y*, and thus the correlation would still hold. Crystal defects and surface irregularities, which are more prominent visually in the larger crystals, are also likely to play a role in the non-uniformity of transition. Indeed, for several of those crystals, the transition can be seen to originate at such defects, which serve as “weak points” with respect to overcoming the lattice energy barrier. The anisotropy of the transition throughout these crystals may explain why larger crystals exhibit more mechanical stress and crystal motion during the measurement (Movies 1–3, supplementary material). As a whole, these results are particularly important for time-resolved crystallographic studies, indicating that molecular synchrony and lattice transition uniformity increase as the size of the crystal decreases. There is, of course, a practical limit on the size of a crystal from which a quality diffraction pattern can be acquired. For studies using an XFEL, which has a typical beam-size of ∼1 *μ*m, crystals with dimensions as small as 1 *μ*m are used routinely. The results presented here demonstrate that crystals larger than >10 *μ*m^2^ show greater spatiotemporal non-uniformity within a 1 *μ*m^2^ ROI. More significant, however, are the implications of the severe spatiotemporal variation with respect to the specific positions (volumes) of the crystal to be illuminated by X-rays. The beam certainly will not hit every crystal in locations with the same conformational homogeneity, which only compounds the issue of the crystal size heterogeneity.

The SSPT is a fundamental phenomenon, and the crystal-size dependence on phase transition homogeneity is of practical importance to X-ray crystallography, especially that using an XFEL to study structural changes of biomacromolecules in crystals that are undergoing a phase transition triggered by ligand binding. Our results demonstrate the necessity of using micro/nanocrystals for such studies and the importance of characterizing SSPTs prior to diffraction using an XFEL. This study, therefore, provides a piece of evidence for crystals used in TRX studies that “smaller is better.”

## MATERIALS AND METHODS

### Solution preparation

Crystallization buffer contained 40 mM sodium cacodylate pH 6.5, 80 mM KCl, 100 mM MgCl_2_, 12 mM spermine tetrahydrochloride, and 32–65% (*v*/*v)* 2-methyl-2,4-pentanediol (MPD). RNA buffer contains 10 mM HEPES pH 7.5, 100 mM KCl, and 0.5 mM EDTA. The stabilization buffer was prepared by mixing a 1:1 volume ratio of crystallization buffer and RNA buffer. The solutions were kept at room temperature.

### Crystal growth on poly-*d*-lysine coated glass bottom dish

To prevent the movement of crystals during SSPT and continuous observation, riboA crystals were directly grown on a positively charged glass bottom surface. A 1:1000 dilution of 0.5 *μ*L of finely crushed crystal seeds was first adsorbed on the surface and incubated for 30 s. The sample was covered tightly with a glass beaker to prevent evaporation. On top of the crystal seeds, 5 *μ*L of equal volume of 7.5 mg/ml gel purified riboA and crystallization buffer (32% MPD) was gently added. The glass bottom with the sample was flipped upside down carefully and placed on the dish cover, which contains 0.7 ml of crystallization buffer (32% MPD). The crystallization set up was tightly wrapped with parafilm and incubated at 22 °C for 12 h.

### Time lapsed PVM recording of SSPT in riboA crystals

Prior to the experiment, the excess of uncrystallized RNA in the crystal sample was removed by thoroughly rinsing with 32% MPD stabilization buffer. The crystals may get dislodged if the washing is rigorous. So, 5–6 ml of 32% stabilization buffer was gently added to and removed from the dish. The dish was then filled with 1.5 ml of stabilization buffer, covered with the glass coverslip, and placed on the microscope stage. Crystals were identified and focused, and the light intensity of the crystal birefringence was adjusted to be optimal by adjusting the angular position of the polarizer. The SSPT was then induced by manually pipetting 1.5 ml of 20 mM adenine in 32% stabilization buffer into the dish, and the video was recorded at a resolution of 2456 × 1842 pixels^2^ with a 200 ms exposure time.

### Time lapsed video processing and analysis

Time-lapsed polarization video microscopy data were visualized, processed, and analyzed with *FIJI* (Schindelin *et al.,* 2012) and custom MATLAB programs. The camera on the polarization video microscope exports raw data as .AVI files. The AVI files were imported into *FIJI* as a z-stack of 16-bit TIFF files and converted to grayscale. Crystal dimensions were measured manually with *FIJI*'s line tool. Regions of interest (ROIs) were selected for analysis, cropped, and saved as a grayscale AVI file. A custom MATLAB (v. 2019 b, Mathworks) program, as described in Ramakrishnan *et al.* (2021), was used to measure the intensity of birefringence as a function of time and its first derivative across the ROI. The pixel resolution of the system was 24.75 nm/pixel, but the theoretical optical resolution of the microscope is on the order of 250 nm. Therefore, square neighborhoods of pixels, defined by their length in pixels (e.g., 1, 2, 4, 8, 10, etc., pixels) were used to calculate the average intensities of superpixels, *I(x,y,t)*. For each superpixel, the *I(x,y,t)* was smoothed using a boxcar average with a window set by the user. The negative time derivative of the intensity, –*dI_S_(x,y,t)/dt*, was calculated from the smoothed intensity traces. Peaks in –*dI_S_(x,y,t)/dt* were identified automatically using the findpeaksxw.m routine (O'Haver, 2020). The position of the *i*th peak in time, *τ_i_(x,y)*, and the width of the peaks, *Δτ_i_(x,y)*, correspond to the *i*th crystal lattice phase transition and the duration of that transition at superpixel *(x,y)*. For each ROI and neighborhood size, a csv file listing all the peak transition times, durations, and x–y positions was exported for further analysis.

Classification of the transition times and durations was performed with MATLAB programs. The transition peaks were identified and quantified using MATLAB's k-means clustering algorithm (Lloyd, 1982, Arthur and Vassilvitskii, 2007) and graphing routines. The algorithm assigns each observation (*τ_i_(x,y)*, *Δτ_i_(x,y)*) to exactly one *k* cluster (*k* is chosen before the algorithm runs) defined by centroids. Scatter plots of (*τ_i_(x,y)*, *Δτ_i_(x,y)*), with each point color-coded by cluster, were generated in MATLAB. The means and standard errors of the means of the *τ_i_(x,y)* and *Δτ_i_(x,y)* within each cluster were calculated in MATLAB and visualized with GraphPad Prism 8.

## SUPPLEMENTARY MATERIAL

See the supplementary material for four figures of image data of various sizes and regions of crystals, two tables of mean T1 and half-width at half-max, and three videos of SSPTs in three crystals of different sizes.

## AUTHORS' CONTRIBUTIONS

S.R. and J.R.S. contributed equally to this work.

## Data Availability

The data that support the findings of this study are available from the corresponding author upon reasonable request.

## References

[1] Anwar, J. and Zahn, D. , Adv. Drug Delivery Rev. 117, 47–70 (2017). 10.1016/j.addr.2017.09.017 28939378

[2] Arthur, D. and Vassilvitskii, S. , in SODA '07: Proceedings of the Eighteenth Annual ACM-SIAM Symposium on Discrete Algorithms (2007) pp. 1027–1035.

[3] Berthou, J. and Jolles, P. , Biochim. Biophys. Acta 336, 222–227 (1974). 10.1016/0005-2795(74)90399-7

[4] Choi, M. C. , Pfohl, T. , Wen, Z. Y. , Li, Y. L. , Kim, M. W. , Israelachvili, J. N. , and Safinya, C. R. , Proc. Natl. Acad. Sci. U. S. A 101, 17340–17344 (2004). 10.1073/pnas.0407925101 15572446 PMC536040

[5] Commins, P. , Desta, I. T. , Karothu, D. P. , Panda, M. K. , and Naumov, P. , Chem. Commun. (Camb) 52, 13941–13954 (2016). 10.1039/C6CC06235K 27711296

[6] Cruz, C. A. V. , Shaban, H. A. , Kress, A. , Bertaux, N. , Monneret, S. , Mavrakis, M. , Savatier, J. , and Brasselet, S. , Proc. Natl. Acad. Sci. U. S. A 113, E820–E828 (2016). 10.1073/pnas.1516811113 26831082 PMC4763786

[7] Dobrianov, I. , Kriminski, S. , Caylor, C. L. , Lemay, S. G. , Kimmer, C. , Kisselev, A. , Finkelstein, K. D. , and Thorne, R. E. , Acta Crystallogr., Sect. D: Biol. Crystallogr. 57, 61–68 (2001). 10.1107/S0907444900014578 11134928

[8] Dunitz, J. D. , Pure Appl. Chem. 63(2), 177–185 (1991). 10.1351/pac199163020177

[9] Gim, M. J. , Beller, D. A. , and Yoon, D. K. , Nat. Commun. 8, 1–9 (2017). 28555628 10.1038/ncomms15453 PMC5459947

[10] Horie, M. , Sassa, T. , Hashizume, D. , Suzaki, Y. , Osakada, K. , and Wada, T. , Angew. Chem., Int. Ed. 46, 4983–4986 (2007). 10.1002/anie.200700708 17526032

[11] Horie, M. , Suzaki, Y. , Hashizume, D. , Abe, T. , Wu, T. D. , Sassa, T. , Hosokai, T. , and Osakada, K. , J. Am. Chem. Soc. 134, 17932–17944 (2012). 10.1021/ja304406c 23039308

[12] Huxley, H. E. and Kendrew, J. C. , Acta Crystallogr. 6, 76–80 (1953). 10.1107/S0365110X5300017X

[13] Khaliullin, R. Z. , Eshet, H. , Kuhne, T. D. , Behler, J. , and Parrinello, M. , Nat. Mater. 10, 693–697 (2011). 10.1038/nmat3078 21785417

[14] Klingl, S. , Scherer, M. , Stamminger, T. , and Muller, Y. A. , Acta Crystallogr., Sect. D: Biol. Crystallogr. 71, 1493–1504 (2015). 10.1107/S1399004715008792 26143921

[15] Kodandapani, R. , Suresh, C. G. , and Vijayan, M. , J. Biol. Chem. 265, 16126–16131 (1990). 10.1016/S0021-9258(17)46197-7 2398048

[16] Kupitz, C. , Basu, S. , Grotjohann, I. , Fromme, R. , Zatsepin, N. A. , Rendek, K. N. , Hunter, M. S. , Shoeman, R. L. , White, T. A. , Wang, D. J. , James, D. , Yang, J. H. , Cobb, D. E. , Reeder, B. , Sierra, R. G. , Liu, H. G. , Barty, A. , Aquila, A. L. , Deponte, D. , Kirian, R. A. , Bari, S. , Bergkamp, J. J. , Beyerlein, K. R. , Bogan, M. J. , Caleman, C. , Chao, T. C. , Conrad, C. E. , Davis, K. M. , Fleckenstein, H. , Galli, L. , Hau-Riege, S. P. , Kassemeyer, S. , Laksmono, H. , Liang, M. N. , Lomb, L. , Marchesini, S. , Martin, A. V. , Messerschmidt, M. , Milathianaki, D. , Nass, K. , Ros, A. , Roy-Chowdhury, S. , Schmidt, K. , Seibert, M. , Steinbrener, J. , Stellato, F. , Yan, L. F. , Yoon, C. , Moore, T. A. , Moore, A. L. , Pushkar, Y. , Williams, G. J. , Boutet, S. , Doak, R. B. , Weierstall, U. , Frank, M. , Chapman, H. N. , Spence, J. C. H. , and Fromme, P. , Nature 513, 261 (2014). 10.1038/nature13453 25043005 PMC4821544

[17] LaFountain, J. R. and Oldenbourg, R. , Mol. Biol. Cell 15, 5346–5355 (2004). 10.1091/mbc.e04-06-0524 15385630 PMC532015

[18] Lloyd, S. P. , IEEE Trans. Inf. Theory 28, 129–137 (1982). 10.1109/TIT.1982.1056489

[19] Mehta, S. B. , Shribak, M. , and Oldenbourg, R. , J. Opt. 15, 094007 (2013). 10.1088/2040-8978/15/9/094007 PMC3834771 24273640

[20] Mnyukh, Y. , *Fundamentals Solid-State Phase Transitions, Ferromagnetism Ferroelectricity*, 2nd ed. ( Directscientific Press, 2010). ISBN: 978-0-615-33972-6.

[21] Mnyukh, Y. V. and Panfilova, N. A. , J. Phys. Chem. Solids 34, 159–159+ (1973). 10.1016/0022-3697(73)90073-5

[22] Nagendra, H. G. , Sukumar, N. , and Vijayan, M. , Proteins: Struct., Funct., Bioinf. 32, 229–240 (1998). 10.1002/(SICI)1097-0134(19980801)32:2<229::AID-PROT9>3.0.CO;2-F 9714162

[23] Nahvi, A. , Sudarsan, N. , Ebert, M. S. , Zou, X. , Brown, K. L. , and Breaker, R. R. , Chem. Biol. 9, 1043 (2002). 10.1016/S1074-5521(02)00224-7 12323379

[24] O'Haver, T. , “Pragmatic introduction to signal processing applications in scientific measurement” (2020).

[25] Pande, K. , Hutchison, C. D. M. , Groenhof, G. , Aquila, A. , Robinson, J. S. , Tenboer, J. , Basu, S. , Boutet, S. , DePonte, D. P. , Liang, M. N. , White, T. A. , Zatsepin, N. A. , Yefanov, O. , Morozov, D. , Oberthuer, D. , Gati, C. , Subramanian, G. , James, D. , Zhao, Y. , Koralek, J. , Brayshaw, J. , Kupitz, C. , Conrad, C. , Roy-Chowdhury, S. , Coe, J. D. , Metz, M. , Xavier, P. L. , Grant, T. D. , Koglin, J. E. , Ketawala, G. , Fromme, R. , Srajer, V. , Henning, R. , Spence, J. C. H. , Ourmazd, A. , Schwander, P. , Weierstall, U. , Frank, M. , Fromme, P. , Barty, A. , Chapman, H. N. , Moffat, K. , van Thor, J. J. , and Schmidt, M. , Science 352, 725–729 (2016). 10.1126/science.aad5081 27151871 PMC5291079

[26] Peng, Y. , Wang, F. , Wang, Z. , Alsayed, A. M. , Zhang, Z. , Yodh, A. G. , and Han, Y. , Nat. Mater. 14, 101–108 (2015). 10.1038/nmat4083 25218059

[27] Perutz, M. F. , Trans. Faraday Soc. 42, B187–B195 (1946). 10.1039/tf946420b187

[28] Pinard, M. A. , Kurian, J. J. , Aggarwal, M. , Agbandje-McKenna, M. , and McKenna, R. , Acta Crystallogr., Sect. F: Struct. Biol. Commun. 72, 573–577 (2016). 10.1107/S2053230X16009286 27380376 PMC4933009

[29] Pogatscher, S. , Leutenegger, D. , Schawe, J. E. , Uggowitzer, P. J. , and Loffler, J. F. , Nat. Commun. 7, 11113 (2016). 10.1038/ncomms11113 27103085 PMC4844691

[30] Porter, D. A. , Easterling, K. E. , and Sherif, M. Y. , *Phase Transformations in Metals and Alloys* ( CRC Press, Boca Raton, FL, 2008).

[31] Ramakrishnan, S. , Stagno, J. R. , Conrad, C. E. , Ding, J. , Yu, P. , Bhandari, Y. R. , Lee, Y. T. , Pauly, G. , Yefanov, O. , Wiedorn, M. O. , Knoska, J. , Oberthur, D. , White, T. A. , Barty, A. , Mariani, V. , Li, C. , Brehm, W. , Heinz, W. F. , Magidson, V. , Lockett, S. , Hunter, M. S. , Boutet, S. , Zatsepin, N. A. , Zuo, X. , Grant, T. D. , Pandey, S. , Schmidt, M. , Spence, J. C. H. , Chapman, H. N. , and Wang, Y. X. , Nat. Commun. 12, 1762 (2021). 10.1038/s41467-021-21838-5 33741910 PMC7979858

[32] Salunke, D. M. , Veerapandian, B. , Kodandapani, R. , and Vijayan, M. , Acta Crystallogr., Sect. B: Struct. Sci. 41, 431–436 (1985). 10.1107/S0108768185002415

[33] Schindelin, J. , Arganda-Carreras, I. , Frise, E. , Kaynig, V. , Longair, M. , Pietzsch, T. , Preibisch, S. , Rueden, C. , Saalfeld, S. , Schmid, B. , Tinevez, J. Y. , White, D. J. , Hartenstein, V. , Eliceiri, K. , Tomancak, P. , and Cardona, A. , Nat. Methods 9, 676–682 (2012). 10.1038/nmeth.2019 22743772 PMC3855844

[34] Schmidt, M. , Adv. Condens. Matter Phys. 2013, 1. 10.1155/2013/167276

[35] Shribak, M. , Sci. Rep. 5, 17340 (2015). 10.1038/srep17340 26611150 PMC4661494

[36] Smets, M. M. H. , Kalkma, E. , Krieger, A. , Tinnemans, I. , Meekes, H. , Vlieg, E. , and Cuppen, H. M. , IUCrJ 7, 331–341 (2020). 10.1107/S2052252520001335 PMC7055385 32148860

[37] Stagno, J. R. , Liu, Y. , Bhandari, Y. R. , Conrad, C. E. , Panja, S. , Swain, M. , Fan, L. , Nelson, G. , Li, C. , Wendel, D. R. , White, T. A. , Coe, J. D. , Wiedorn, M. O. , Knoska, J. , Oberthuer, D. , Tuckey, R. A. , Yu, P. , Dyba, M. , Tarasov, S. G. , Weierstall, U. , Grant, T. D. , Schwieters, C. D. , Zhang, J. , Ferré-D'Amaré, A. R. , Fromme, P. , Draper, D. E. , Liang, M. , Hunter, M. S. , Boutet, S. , Tan, K. , Zuo, X. , Ji, X. , Barty, A. , Zatsepin, N. A. , Chapman, H. N. , Spence, J. C. H. , Woodson, S. A. , and Wang, Y. X. , Nature 541, 242–246 (2017). 10.1038/nature20599 27841871 PMC5502819

[38] Suga, M. , Shimada, A. , Akita, F. , Shen, J. R. , Tosha, T. , and Sugimoto, H. , Biochim. Biophys. Acta, Gen. Subj. 1864, 129466 (2020). 10.1016/j.bbagen.2019.129466 31678142

[39] Taniguchi, T. , Sato, H. , Hagiwara, Y. , Asahi, T. , and Koshima, H. , Commun. Chem. 2, 10 (2019).

[40] Tenboer, J. , Basu, S. , Zatsepin, N. , Pande, K. , Milathianaki, D. , Frank, M. , Hunter, M. , Boutet, S. , Williams, G. J. , Koglin, J. E. , Oberthuer, D. , Heymann, M. , Kupitz, C. , Conrad, C. , Coe, J. , Roy-Chowdhury, S. , Weierstall, U. , James, D. , Wang, D. J. , Grant, T. , Barty, A. , Yefanov, O. , Scales, J. , Gati, C. , Seuring, C. , Srajer, V. , Henning, R. , Schwander, P. , Fromme, R. , Ourmazd, A. , Moffat, K. , Van Thor, J. J. , Spence, J. C. H. , Fromme, P. , Chapman, H. N. , and Schmidt, M. , Science 346, 1242–1246 (2014). 10.1126/science.1259357 25477465 PMC4361027

[41] Vrabioiu, A. M. and Mitchison, T. J. , Nature 443, 466–469 (2006). 10.1038/nature05109 17006515

[42] Wang, W. J. , Miller, J. P. , Pannullo, S. C. , Reinhart-King, C. A. , and Bordeleau, F. , J. Biophotonics 11, e201800008 (2018). 10.1002/jbio.201800008 29931742 PMC6226342

[43] Yoon, D. K. , Choi, M. C. , Kim, Y. H. , Kim, M. W. , Lavrentovich, O. D. , and Jung, H. T. , Nat. Mater. 6, 866–870 (2007). 10.1038/nmat2029 17934466

